# Wu-Teng-Gao External Treatment Improves Th17/Treg Balance in Rheumatoid Arthritis

**DOI:** 10.1155/2022/5105545

**Published:** 2022-01-21

**Authors:** Xueming Yao, Qiuyi Wang, Changming Chen, Ping Zeng, Lei Hou, Jing Zhou, Ying Huang, Wukai Ma

**Affiliations:** ^1^Department of Rheumatology and Immunology, The Second Affiliated Hospital of Guizhou University of Traditional Chinese Medicine, Guiyang 550001, China; ^2^Guizhou Medical University, Beijing Road, Guiyang, Guizhou Province 550004, China

## Abstract

Rheumatoid arthritis (RA) represents the consequence of an immune response of the body's immune system attacking healthy cells. This chronic inflammatory disorder has complicated pathogenesis. Traditional Chinese medicine (TCM) is well recognized as an effective therapy in treating RA and has been widely applied for centuries. Wu-Teng-Gao (WTG) is used as a representative natural herb formula in RA treatment in China, while its mechanisms are to be fully clarified. The present study attempted to explore mechanisms of WTG on RA treatment in a network pharmacological approach and verified using experiments in vitro. Following the establishment of a rat model of collagen-induced arthritis (CIA), WTG was applied externally on the metapedes of rats. HE staining was subsequently performed to visualize the pathological changes of synovium and bone. Simultaneously, flow cytometry was conducted to detect the cell ratio of T helper 17 (Th17) and Regulatory T cells (Treg) in splenic lymphocytes. Additionally, ELISA, qRT-PCR, and Western blot assays were adopted to determine expressions of RA-related factors in joints and serum. Results of network pharmacological analysis suggested that Th17 cell differentiation might serve as a potential signaling pathway of WTG therapy for RA. Animal experiments demonstrated that WTG ameliorated the articular inflammation and effectively inhibited the destruction of articular cartilage, and decreased Th17 and Treg cell ratios in CIA rats. Furthermore, WTG also greatly suppressed relevant levels of inflammatory cytokines (IL-17, TNF-*α*, IL-1, and IL-6) and RNAKL, whereas it elevated expressions of anti-inflammatory cytokines IL-10 and TGF-*β*. Our results confirmed that WTG might improve the imbalance of Th17/Treg cells in CIA animals through differentiation regulation, thus alleviating joint inflammation and bone destruction.

## 1. Introduction

Rheumatoid Arthritis (RA) has been well recognized as a systemic and chronic autoimmune disorder. The principal characteristics include the proliferation of synovial cells, infiltration of inflammatory cells, as well as destruction in articular cartilage and bones, thus resulting in joint deformity and disability [1]. The RA pathogenesis is intricate and unclear, which can be triggered by genetic factors, environmental factors, and aberrant immune systems [2]. As one subset of T cells [3], Th17 cells play an essential role in proinflammation, and when in excess, such cells contribute to autoimmunity and tissue damage. The other subset of T cells is Treg cells, which are antagonistic, and once they fail, the same diseases occur [3]. The Th17/Treg ratio is lower in healthy control than that of RA patients, especially the active RA patients [4]. Th17/IL-17 axis is of great significance in local inflammation and bone destruction of RA joints [5, 6]. Exosomes contain multiple proteins and nucleic acids, which are involved in T cell activation, antigen expression, intracellular signal transduction, inflammatory response, bone destruction, microvascular dysplasia, and other aspects in the pathogenesis of RA [7–9]. A recent report has shown that exosomes can regulate Thl7/Treg imbalance and inhibit the Th17/IL17 axis [10].

TCM is widely used in China and is effective in treating RA [11]. RA, in light of TCM theory, is considered to be an “impediment disease” (also Bi syndrome), as a consequence of invasion by cold, heat, wind, or dampness pathogens into the meridian channels [12]. Following the principles of TCM theory, dampness and cold elimination and blood circulation promotion contribute to treating severe RA [13]. The current drug administration of RA treatment includes steroidal and non-steroidal anti-inflammatory medication, disease-modifying antirheumatic drugs, and biological preparations in clinical practice [14]. Unfortunately, the previously mentioned medications improve the symptoms slowly and even have certain serious adverse reactions after long-term administration [15]. A meta-analysis has indicated that the treatment protocols that integrated TCM and Western medicines can achieve both effective and satisfactory results in treating RA [16]. The TCM herb *Tripterygium Wilfordii, Caulis Sinomenii*, and *Periploca Forrestii* have been proved effective in alleviating RA progression [17–19]. Triptolide, a component of *Tripterygium Wilfordii* displays an immunosuppressive effect on the RA animal model by downregulating Th17 cells [20]. TCM can realize the overall regulation of body functions through multifaceted and multi-target mechanisms, and it produces a remarkable curative effect on the treatment of RA [11]. However, the underlying mechanism by which TCM helps to alleviate RA remains unknown, thereby hindering its further clinical application.

Our preliminary clinical study confirmed that WTG had a good anti-RA effect in external treatment. The results of the network pharmacological approach indicated that WTG might be of great importance in the Th17 cell differentiation pathway. Based on this, we proposed the hypothesis that the WTG could relieve local joint inflammation and bone destruction by regulating Exo to inhibit the Th17/IL-17 signal axis and relieve systemic inflammation by mediating Th17/Treg balance. The present research explored the anti-arthritis effect using a CIA rat model treated with WTG and the possible mechanism of Th17/Treg balance through exosomes, expecting to offer an experimental foundation for RA treatment in clinical application.

## 2. Material and Method

### 2.1. Analysis of Molecular Networks and Signaling Pathways

Based on the principle of network pharmacology, the main components and action targets of compound WTG were predicted to offer a research basis for further study of its mechanism of action. The effective active components and related targets of Da-Xue-Teng (*Sargentodoxae Caulis*), Ji-Xue-Teng (*Spatholobus Suberectus*), Qing-Feng-Teng (*Sinomenium Acutum*), Lei-Gong-Teng (*Tripterygium Wilfordii*), and Hei-Gu-Teng (*Periploca Forrestii* Schltr.) were retrieved via the TCMSP platform (http://lsp.nwu.edu.cn/tcmsp.php), and the genes related to RA were selected from the disease database GeneCard (https://www.genecards.org). Subsequently, the diagram of protein protein interaction (PPI) was constructed using the String website (https://string-db.org/). Analysis of GO and KEGG pathway enrichment of key targets was performed, and the top 30 terms with the lowest *P* values were selected for visualization analysis.

### 2.2. Rat CIA Modeling

Animal experiments were approved and performed in accordance with the guidelines of Committee on the Ethics of Animal Experiments of The Second Affiliated Hospital of Guizhou University of Traditional Chinese Medicine, China (No. KYW2019001). A total of 70 SPF grade rats aged 6–8 weeks, weighing approximately 150–180 g were provided by Chongqing Ensiweier Biotechnology Co. Ltd. All animals were given adaptive feeding for one week and marked with numbers. The animal house was provided with an alternative of 12 h–12 h day and night. Water and diets were given at will. The temperature was maintained at 23–25°C.

The laboratory rats were randomly classified into 7 groups: a blank group, a model group, an IL-17 blocking group, a diclofenac diethylamine emulgel (DDE) control group, a high-dose Wu-Teng-Gao group, a medium-dose Wu-Teng-Gao group, and a low-dose Wu-Teng-Gao group, with 10 rats in each group. In the treatment group, high (0.45 g/paw), medium (0.3 g/paw), and low dose (0.15 g/paw) of Wu-Teng-Gao were externally applied on metapedes of the tested rats, respectively. The blank group and model group were fed normally and given an equal dosage of Vaseline for external application. The positive control group was given diclofenac diethylamine emulsion (20 g/tube, produced by Beijing Novartis Pharmaceutical Co., Ltd.), 70 *μ*L/100 g.

The CIA rat model of arthritis is characterized by similar clinical, histopathological, and immunological changes to the human, and it is the animal model of bone destruction commonly used internationally [21, 22]. Collagen II (SolarBio, China) was dissolved by supplementing 0.1 mol/L acetic acids to prepare a 2 mg/mL solution, stirred to be dissolved, and placed in a refrigerator at 4°C overnight. Freund's incomplete adjuvant at the same volume was supplemented, mixed, and emulsified into type II collagen emulsion [23]. The rat model was subsequently injected with a 0.1 mL emulsifier into the tail root intradermally to induce inflammation. The stable CIA model was reproduced, and corresponding drug intervention was initiated to confirm successful modeling. The model assessment followed the below-described criteria: 0 point represented no erythema or swelling; 1 point represented slight erythema or swelling of one toe; 2 points represented erythema or swelling of more than one toes; 3 points represented erythema and swelling of the ankle or wrist; and 4 points represented all erythema and swelling of the toes and ankles or fingers and wrists [24] and failure to bend the ankles or wrists.

### 2.3. Drug Intervention

Miao medicine Wu-Teng-Gao consists of Hei-Gu-Teng, Ji-Xue-Teng, Qing-Feng-Teng, Da-Xue-Teng, and Lei-Gong-Teng. The preparation was provided by the Pharmacy Department of The Second Affiliated Hospital of Guizhou University of Traditional Chinese Medicine. Doses of the high, medium, and low treatment groups were calculated by crude drug dose. The medium dose was the same as the crude drug used in clinical patients (the drug volume was about 30 g/paw), according to the conversion coefficient table of drug dose for adults and animals [25]. Drug administration was initiated on day 7 of the modeling. All groups were fixed with nonwoven fabric (patch backing layer), once per day. The course lasted 4 weeks. The IL-17 blocking group was injected with an IL-17 neutralizing antibody, 50 *μ*g/time/rat, once every 3 d. The weight of the rats was measured once a week. On day 28 after administration, the rats were put to death by intraperitoneally injecting 100 mg/kg pentobarbital sodium. Blood samples were obtained from the abdominal aorta and serum samples were collected by centrifugation [26]. The synovial tissues and joint tissues were obtained by removal of the skin on the knee joint and ankle joint of both hind limbs, part of which was fixed with 4% paraformaldehyde, and the remaining were cryopreserved in a refrigerator at −80°C for subsequent detection of various indicators in further experiments.

### 2.4. HE Staining

The tissues were immersed in a fixation solution containing 4% paraformaldehyde 24 h and prepared into sections at 5 *μ*m thickness. Paraffin sections were baked in an oven set at 60°C for 1 h, dewaxed with xylene, and followed by gradient alcohol hydration. Then they were rinsed with double distilled water for 3 times, 5 min each time. After staining with hematoxylin solution for 5 min, differentiation was performed using hydrochloric acid and alcohol for 2–3 s, saturated lithium carbonate solution turned blue for 1–2 min. After washing with running water for 10 min, drop staining was performed using eosin solution for 1–3 min and washed with running water for 10 min. Conventional gradient alcohol was employed for dehydration and transparency and slice sealing.

### 2.5. Primary Isolation and Culture of Splenic Lymphocytes

The spleens of the previously described rats were taken after treatment according to the experiment grouping. After surface disinfection, the spleens were dissected, cut into pieces with surgical shears, homogenized manually in an ice bath, and passed through a stainless 200 mesh sieve [27]. The single-cell suspension in the Petri dishes was collected. The operation procedures were conducted following the instructions of the rat spleen lymphocyte separation medium kit (Solarbio, China). An addition of 3 mL separation solution was supplemented to a 15 mL centrifuge tube, and an equal volume of single-cell suspension was added. Centrifugation was performed at room temperature with a horizontal rotor at 500–1000*g* for 30 min. After centrifugation, a distinct stratification was visualized: the superficial layer was the diluent layer, followed by a layer of lymphocytes presenting a ring of milky white. The third layer was a transparent separation liquid layer. And the fourth was the red blood cell layer. The white layer cells were carefully aspirated into a clean 15 mL centrifuge tube, washed with 10 mL PBS, and centrifugated at 250*g* for 10 min. The supernatant was removed, and the cells were resuspended with 5 mL PBS or cell cleaning solution, centrifuged at 250*g* for 10 min, and resuspended for later use.

### 2.6. Flow Cytometry Detection

Splenic single-cell suspension 300 *μ*L was taken from each sample of each group, and PMA (final concentration 50 *μ*g/L), 1 *μ*L ionomycin (final concentration 1 mg/L), and 1 *μ*L monenomycin (final concentration 1 mg/L) were added and mixed well. The cells were incubated at 37°C in a 5% CO_2_ incubator for 6 h, and the cell suspension was resuspended by oscillation once during the process. After incubation, the cell suspension was taken and washed with precooled PBS to remove residual irritants. FITC-labeled anti-rat CD4 antibody 1 *μ*L was added. After incubation at 4°C for 60 min in the dark 900 *μ*L RBC lysate was added, then incubated at room temperature for 7 min avoiding light, and washed with PBS twice. Following centrifugation at 3000 r/min using a high-speed micro-centrifuge for 3 min, the solution was washed with PBS once and the supernatant was discarded. The cells were resuspended in the residual fluid. Samples of each group were added with 1 mL of pre-diluted Foxp3 detection drilling/fixation concentrate three times in advance, rotated, mixed well, and followed by incubation at 4°C for 60 min in darkness. Following a cycle of washing with 2 mL diluted 1× permeation working solution, the cells were centrifuged at 3000 r/min for 3 min, the supernatant was discarded, and washing was repeated once. The supernatant was discarded and the cells were resuspended in the residual fluid. Antibodies anti-rat Foxp3 0.5 *μ*L and anti-rat IL-17A 1 *μ*L were added to each sample, and 1 *μ*L of IgG2a Isotype Control (2A3) was set up and incubated at 4°C for 60 min in the dark. After that, 2 mL of permeation working solution was added to wash the cells twice, and the supernatant was discarded. 500 *μ*L cold PBS was added to resuspend cells. Cells were detected by flow cytometry immediately.

### 2.7. Real-Time Fluorescence Quantitative PCR (qRT-PCR)

RNA was extracted from rat joint tissues and spleen tissues using RNAiso Plus (Takara, Japan). Goldenstar™ RT6 cDNA Synthesis Kit (Beijing, Qingke) was employed to synthesize cDNA. The real-time fluorescence quantitative processes and procedures were followed by the instructions of the 2 × T5 Fast qPCR Mix (SYBR Green I) (Tsingke Biological Technology, Beijing). qPCR amplification procedures were designed as following: 95°C for 30 s with 40 cycles at 95°C for 5 s, 55°C for 30 s, and 72°C for 30 s. Gene expression was relative to GAPDH. Primer sequences were listed (Table 1).

### 2.8. Western Blot

Joint tissues and spleen tissues of each group were added with lysate containing PMSF and protease inhibitor Cocktail RIPA (Beyotime, China) to obtain total protein [28]. Total proteins 500 *μ*g were extracted from each sample and 5 × SDS loading buffer (China, Jalase) were mixed at the ratio of 4 : 1 to set the proteins at a concentration of about 3.3 *μ*g/*μ*L, and followed by denaturation using a metal bath heated at 100°C for 6 min. The denatured total proteins of 60 g were selected for sample loading. The protein was transferred to a PVDF (Amersham, Germany) membrane. The membrane was removed, washed in TBST for 1 min, and then sealed with 5% skimmed milk blocking buffer at room temperature for 1 h. After sealing, it was washed 3 times using TBST, 5 min each time. The primary antibody was diluted with A primary antibody diluent at 1 : 1000 and incubated overnight at 4°C, followed by three cycles of washing with TBST, 10 min each time. Secondary antibody was diluted at 1 : 2000 concentration with the blocking buffer, incubated for 1 h, then rinsed with TBST for 3 times, 10 min each time. The ECL exposure solution (Thermo, USA) was evenly mixed with liquid A and B at a 1 : 1 rate and evenly spread on the full film surface. After reaction for 1 min, it was loaded in the exposure instrument for detection.

Procedures were performed following the instructions of reagent kits for IL-1, IL-10, IL-17, IL-6, TNF-*α*, and TGF-*β* ELISA (Ruixin Biology). Briefly, the reagent kits were balanced at room temperature, and standard wells and sample wells were set. The addition of 50 *μ*L standard solution of different concentrations was supplied to each standard well, and a 10 *μ*L sample was initially supplied to the sample wells and subsequently 40 *μ*L sample dilution for testing. Detection antibody labeled by horseradish catalase 100 *μ*L was supplemented to each standard solution well and sample well, respectively. Following the blocking of well plate with a sealing membrane, incubation was performed at 37°C for 60 min. After incubation, the liquid in wells was discarded and a washing solution was added, standby for 1 min, and discarded with 5 repeats. Following the addition of substrate A and B 50 *μ*L to each well, incubation was performed at 37°C for 15 min in the dark. Termination solution at a quantity of 50 *μ*L was added to each well after incubation. OD values were measured at 450 nm wavelength within 15 min.

### 2.9. Statistical Analysis

Data analysis was performed using the SPSS 23.0 software. GraphPad Prism 8.0 was employed for plotting. Mean comparison of multiple groups was subjected to one-way ANOVA. The Tukey method was employed for pairwise comparison. The results obtained from all experiments were expressed as the mean ± SD. The value of *P* < 0.05 was regarded as statistically significant.

## 3. Results

### 3.1. Results of Network Pharmacological Analysis

**Figure 1:**
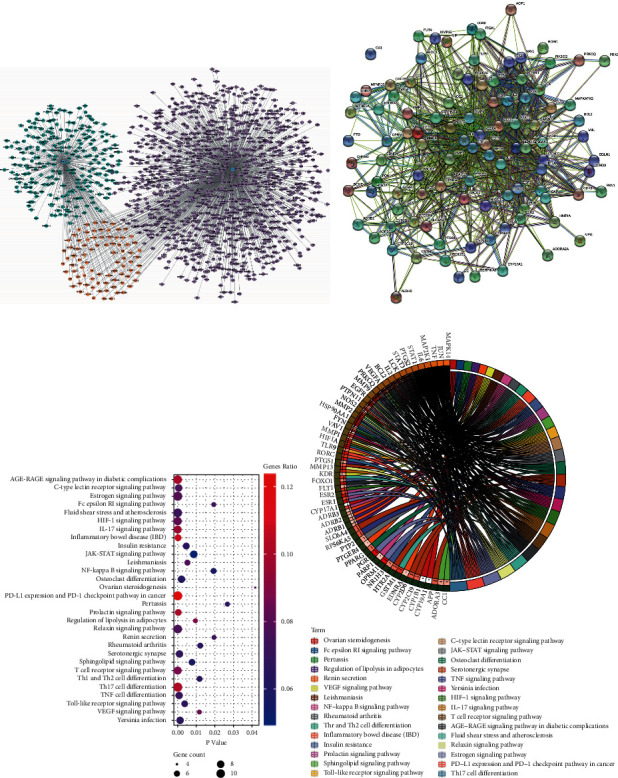
Network pharmacological analysis of Wu-Teng-Gao (WTG) on rheumatoid arthritis (RA). (a) Network of WTG-related targets and RA-related targets. Green represents the WTG-related targets, purple represents the RA-related targets, and yellow represents the common targets between them. (b) Protein protein interaction (PPI) network diagram of overlapped targets. (c) The top 30 GO terms of common targets. The *Y* axis represents GO terms on the left, and the *X* axis represents *P* values. (d) The top 30 KEGG pathway of the overlapped targets. The name of the signaling pathways are presented on the right, and the targets are on the left. The darker the left inner circle, the greater the *P* values of the corresponding gene pathways.

A total of 402 targets of WTG were collected from the TCMSP database and 1165 genes related to RA were retrieved from the Gene database (Figure 1(a)). RA and WTG had 85 overlapped targets and the PPI network showed a correlation between them (Figure 1(b)). The top 30 signaling pathways of GO analysis were focused on Th17 cell differentiation, IL-17 signaling pathways, cellular immune response, inflammatory response, and cytokine signaling (Figure 1(c)). Further KEGG analysis showed that the Th17 cell differentiation signaling pathway was identified as the top one common signaling pathway (Figure 1(d)).

### 3.2. Histopathological Changes in CIA Rats Treated by WTG

**Figure 2:**
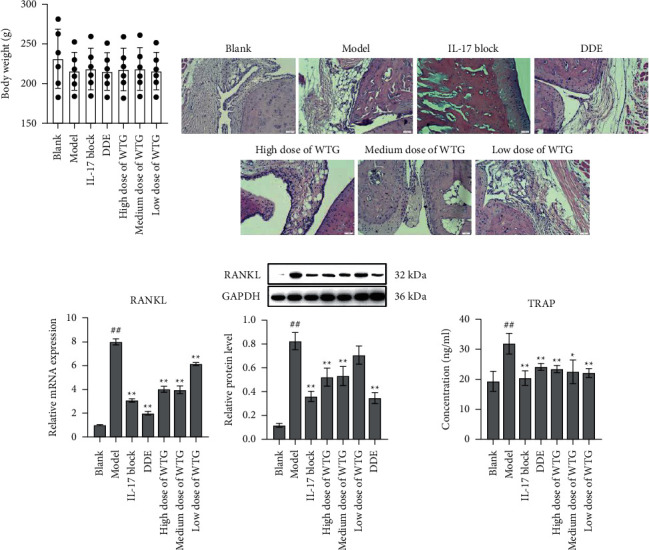
WTG improved bone destruction in CIA rats. (a) Changes in rat body weight after treatment with high (0.45 g/paw), medium (0.3 g/paw), and low doses (0.15 g/paw) of WTG in CIA rats. The body weight was measured before modeling. Drug administration was initiated on day 7 of the CIA modeling, and body weight was measured once a week for 4 consecutive weeks. (b) Histopathological changes in joint tissues after 4 weeks of drug administration. Scale bar = 50 *μ*m. (c) RANKL mRNA levels in joint tissues. (d) RANKL protein levels in joint tissues were tested by Western blot. (e) TRAP levels in serum by ELISA kit. Protein quantification was determined by Image J Mean comparison of multiple groups was subjected to one-way ANOVA with Turkey test. *n* = 10, ^##^*P* < 0.01, compared to Blank; ^*∗∗*^*P* < 0.01, compared to Model. DDE, diclofenac diethylamine emulsion agent.

Throughout the experiment, body weight of the normal group was heavier than that of the model group and drug treatment group (Figure 2(a)). To evaluate the impact of WTG on CIA-induced joint pathological injury and inflammation, HE staining was performed using knee joint tissues. In the normal group, the surface of articular cartilage was smooth and integrated, and there was neither articular cartilage destruction nor bone destruction. Infiltration of synovial inflammatory cells, proliferation and disorder of synovial cells, and proliferation of capillaries were observed in the model group. Superficial cartilage appeared uneven, defective, and exfoliated. Furthermore, severe damages in both articular cartilage and bone were visualized. The number of inflammatory cells decreased in the IL-17 blocking group and DDE group. Meanwhile, the surface articular cartilage was relatively smooth, and cartilage and bone destruction were greatly alleviated. In the low-dose WTG group, the cartilage surface remained uneven, and the bone was destroyed seriously with disarranged synovial cells. The number of inflammatory cells was declined in the medium and high doses WTG groups. The surface of articular cartilage was slightly rough presenting an integrated structure, and no evident destruction of cartilage or bone tissues was visualized (Figure 2(b)).

### 3.3. Effect of WTG on the Bone Destruction

Further, the expression of RANKL in joint tissues was subjected to qRT-PCR and Western blot assays, and the influence of WTG on bone destruction was explored in CIA rats. In comparison to the blank group, RANKL mRNA and protein levels were markedly increased, whereas RANKL levels were markedly lower in WTG-treated, IL-17 blocker, and DDE groups. Although WTG could decrease RANKL levels, it remained higher than the blank group (Figures 2(c) and 2(d)). Compared with the model group, TRAP levels in peripheral blood of CIA rats were increased significantly in the WTG group (Figure 2(e)). The data indicated that WTG could suppress the expression of RANKL and inhibit osteoclast differentiation, affecting osteoclast function, and thereby reducing bone destruction.

### 3.4. Effect of WTG on Th17/Treg Cells

**Figure 3:**
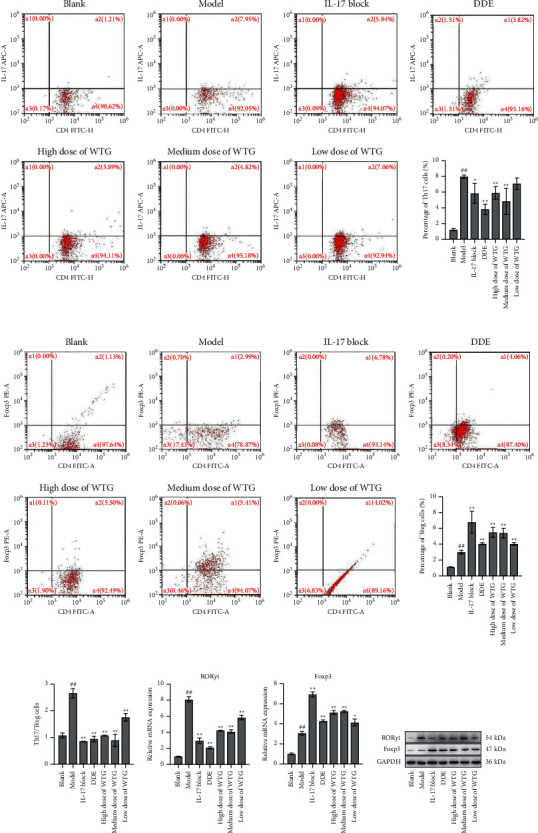
WTG decreased the ratio of Th17/Treg cells in CIA rats. Th17 cell ratio (a) and Treg cell ratio (b) in CD4+ cells after treatment with high (0.45 g/paw), medium (0.3 g/paw), and low doses (0.15 g/paw) of WTG. (c) Ratios of Th17/Treg cells. (d) ROR*γ*t and Foxp3 mRNA levels in the spleen tissue. (e) ROR*γ*t and Foxp3 protein levels in the spleen tissue. Mean comparison of multiple groups was subjected to one-way ANOVA with Tukey test. *n* = 10, ^##^*P* < 0.01, compared to Blank; ^*∗*^*P* < 0.05,  ^*∗∗*^*P* < 0.01, compared to Model. DDE, diclofenac diethylamine emulsion agent.

Expressions of Th17 and Treg cells in splenic lymphocytes were determined using flow cytometry. Compared with the blank group, Th17 cells in the model group increased significantly. The proportion of Th17 cells in the model group declined as the doses increased in the WTG and the IL-17 blocker groups, and the diclofenac diethylamine emulsion (DDE) control group were also markedly reduced (Figure 3(a)). Compared with the blank group, Treg cells were markedly elevated in the model group, whereas those in the WTG, IL-17 blocker, and DDE groups were increased markedly compared with the model group (Figure 3(b)). The ratio of Th17/Treg cells in the model group was significantly increased compared with the blank group. Treatment with different doses of WTG significantly decreased the ratio of Th17/Treg cells, while efficacy in the low-dose group was the least satisfactory (Figure 3(c)). Results of qRT-PCR and Western blot showed that treatment with different doses of WTG significantly decreased ROR*γ*t levels while markedly increased Foxp3 levels in the spleen tissue (Figures 3(d) and 3(e)). The results indicated that WTG could improve the balance of Th17/Treg cells in CIA rats. WTG produced similar effects on both IL-17 blockers and DDE.

### 3.5. Effect of WTG on Inflammatory Response

**Figure 4:**
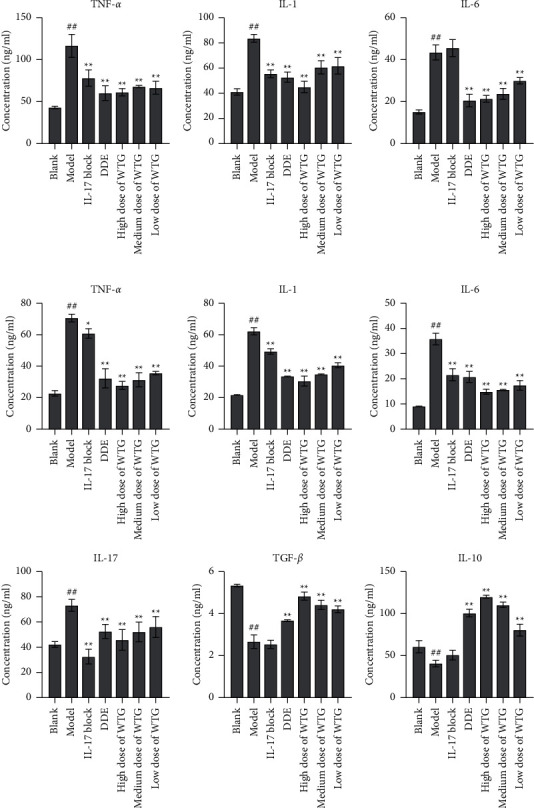
WTG inhibited pro-inflammatory cytokines in CIA rats. (a) Levels of TNF-*α*, IL-1, and IL-6 in the joint tissues were measured by ELISA assay. (b) Levels of TNF-*α*, TGF-*β*, IL-1, IL-6, IL-10, and IL-17 in the serum were measured by ELISA assay. Mean comparison of multiple groups was subjected to one-way ANOVA with the Tukey test. *n* = 10, ^##^*P* < 0.01, compared to Blank; ^*∗*^*P* < 0.05,  ^*∗∗*^*P* < 0.01, compared to Model. DDE, diclofenac diethylamine emulsion agent.

Expressions of TNF-*α*, IL-1, and IL-6 in joint tissues and those of IL-17, TNF-*α*, IL-1, IL-6, IL-10, and TGF-*β* in peripheral blood were determined by ELISA assay. The results revealed that levels of TNF-*α*, IL-1, and IL-6 in joint tissues were increased substantially in the model group when compared to the blank group, whereas those were obviously reduced following WTG administration (Figure 4(a)). In serum, the levels of anti-inflammatory cytokines IL-10 and TGF-*β* in WTG groups were higher than in the model group. The levels of pro-inflammatory factors IL-1, IL-17, TNF-*α*, and IL-6 in WTG groups were lower than in the model group. Moreover, WTG efficacy on these factors was improved with the increase of doses (Figure 4(b)).

## 4. Discussion

RA represents a frequently encountered chronic autoimmune disorder [29]. In TCM, it has been classified into “Bi syndrome” as a result of evil factor invasion, including wind, cold, dampness, and heat, obstructing joints, and causing pains [11]. This disease presently cannot be eradicated clinically but alleviated through some medication treatment and external application therapy to minimize joint pain and maintain the functions [30]. Numerous clinical trials have testified that TCM contributes to RA treatment with definite efficacy, safety, and less toxic side effects [31–33]. WTG is characterized by dispelling wind, dredging meridians, tonifying qi, and activating blood circulation, thereby benefiting tendon relaxation, collateral activation, blood stasis elimination, and pain relief. As a characteristic external therapy, the external application of TCM can effectively regulate the body balance by local drug penetration to dispel nearby evil and treat internal disorder via an external application [34]. Previous studies have reported that multiple Chinese herbal medicines and extracts in WTG formula produce vital effects on pain improvement in RA patients. For example, Qing-Feng-Teng (*Sinomenium Acutum*), Lei-Gong-Teng (*Tripterygium Wilfordii*), and Hei-Gu-Teng (*Periploca Forrestii Schltr.*) are effective in anti-inflammation and RA treatment [18, 35, 36]. Despite several clinical studies that have been conducted, the molecular mechanism by which WTG alleviates RA progression remains poorly understood. The advances of network pharmacology is new technique for TCM research, and the systematic and integrated research concept is in agreement with characteristics of TCM compounds including multi-components, multi-efficacy, and synergistic actions [37]. Currently, network pharmacology has achieved satisfactory results in the pharmacological mechanism of TCM [38]. The network pharmacological analysis results of WTG indicated that RA treatment might mainly act on biological processes of Th17 cell differentiation, IL-17 signaling pathway response, immune response, and inflammatory response. The present study proposed that external application of WTG might alleviate local joint inflammation and bone destruction in CIA rats by inhibiting the Th17/IL-17 signaling axis, thereby mediating Th17/Treg balance to relieve systemic inflammation through network pharmacological analysis.

Treg and Th17 participate in the occurrence and development of autoimmune diseases, tumors, and infections [39]. Treg is of great significance in autoimmune disorders, and it suppresses the secretion of antibodies produced by inflammatory cytokines and maintains the internal immune environment balance of the body [39]. In contrast to Treg, Th17 can promote the development of autoimmune diseases and mediates pro-inflammatory responses [39]. Additional research has implicated that Th17/Treg balance is the key factor to maintain the presence of RA immune homeostasis [40]. The ratio of Th17/Treg cells presenting in the peripheral blood of RA patients is markedly increased and correlated with disease activity [41]. The controlled Th17/Treg ratio assists in alleviating RA and alleviates disease symptoms, and it also plays a key role in treating autoimmune diseases namely RA [42]. The present study detected spleen lymphocytes in CIA rats by flow cytometry assays and the findings indicated that both medium and low doses of WTG therapy could reduce the Th17/Treg ratio and restore to a certain level, whereas the effect of low-dose group was less effective. Transcription factors are well recognized to be pivotal in cell differentiation and development. ROR*γ*t acts as an essential transcription factor in Th17 cells, and its deletion leads to a reduced Th17 cell population and a decreased incidence of autoimmune diseases. Foxp3 is currently recognized as the most sensitive marker and a specific transcription factor of Treg cells. Foxp3 depletion inhibits the differentiation of Treg cells [43]. Foxp3 and ROR*γ*t antagonize each other, and their balance can determine the direction of initial CD4^+^ T cell differentiation to Th17 or Treg cells after antigen stimulation, which affects RA occurrence and development [44]. Our results showed that WTG treatment elevated the expression level of Foxp3 and decreased that of ROR*γ*t in the spleen tissues of CIA rats. The results further confirmed that WTG could mediate the expressions of Foxp3 and ROR*γ*t, thereby regulating the differentiation of Th17/Treg cells and restoring their ratios to a certain extent.

Th17 mostly secretes the inflammatory mediator IL-17, representing an important pro- and pre-inflammatory cytokine and a fine-tuning factor of the inflammatory response [45]. It also participates in the inflammatory response and progressive damage of RA in multiple ways [46]. IL-17 can induce synovial fibroblasts, osteoclasts, and macrophages to secrete pro-inflammatory cytokines TNF-*α*, IL-1, and IL-6, which aggravates joint inflammation and causes the occurrence and development of RA [46]. Conversely, Treg cells can secrete anti-inflammatory cytokines IL-10 and TGF-*β*, inhibit T cells and antigen presentation cells, and exert an immunosuppressive effect by reducing the proinflammatory cytokines secretion [47]. TGF-*β* influences the differentiation of Th17 cells and participates in regulating Treg functions, especially to maintain the balance of Th17/Treg [48]. The expression levels of TNF-*α*, IL-1, and IL-6 in the joint tissues of the CIA model group in the current study were markedly higher than those of the blank group. Following external application using WTG, the expressions of TNF-*α*, IL-1 and IL-6 were largely downregulated compared with the model group. Serum detection results were similar, and external application of WTG to CIA rats reduced the expressions of TNF-*α*, IL-1, IL-6, and IL-17 in a dose-dependent manner. Furthermore, WTG treatment elevated the expression levels of IL-10 and TGF-*β* in the serum of CIA rats. The previously described results indicated that WTG treatment in CIA rats could effectively reduce the proinflammatory cytokines IL-17 secreted by Th17 cells and TNF-*α*, IL-1, and IL-6 induced by Th17 cells, thereby affecting the differentiation of Th17 and Treg and alleviating inflammatory responses of CIA rats.

The principal clinical manifestations of RA are multiple joint erosive synovitis, and the major pathological changes include synovial tissue hyperplasia and the progressive destruction of articular cartilage and bones [6]. The results of HE staining of joint tissues demonstrated that inflammatory cells were reduced in the medium and high dose WTG groups, and the surface of articular cartilage was slightly rough with complete structure in the absence of evident damage of cartilage and bone tissue, suggesting that WTG greatly improved local joint inflammation and bone damage in CIA rats. Previous studies have confirmed that RANKL is highly expressed in RA patients and promotes synovitis and bone destruction in RA [49]. IL-17 can upregulate the expression of RANK, the precursor of osteoclasts, enhance the RANKL signaling pathway and facilitate the differentiation of osteoclasts by inducing RANKL expression [50]. Okamoto and Takayanagi have shown that Treg cells can reduce osteoclast formation by inhibiting the RANKL pathway [51]. Furthermore, IL-10 and TGF-*β* secreted by Treg can downregulate the expression of RANKL and reduce bone injury [51]. Our results indicated that WTG treatment could substantially reduce the expression of the RANKL gene and protein in the joint tissues of CIA rats, suggesting that WTG might reduce the level of RANKL by inhibiting IL-17 and upregulating IL-10 and TGF-*β*, thereby improving local joint inflammation and bone destruction.

## 5. Conclusion

Taken together, this study elucidated that WTG could reduce expressions of pro-inflammatory factors IL-17, TNF-*α*, IL-1, and IL-6, and increase expressions of anti-inflammatory factors IL-10 and TGF-*β*. These results indicated that WTG might improve the Th17/Treg imbalance of immune cells in CIA arthritis rats, thereby contributing to alleviating joint inflammation and bone destruction. In future research, we will further explore the molecular mechanism of key monomers in WTG, and use technologies such as molecular docking and microscale thermophoresis to further clarify the direct regulation of WTG.

## Figures and Tables

**Table 1:** The primers used for qRT-PCR.

Primers	Sequences
RANKL-F	TACGGCAAGTACCTGCGCG
RANKL-R	CCAGGAGCGCCAGGAACATGA
ROR*γ*t-F	GCAAGTCCTACCGAGAGACGT
ROR*γ*t-R	CCTCTGGTAGCTGGTCACCT
Foxp3-F	TCCCACAAGCCAGGCTGATC
Foxp3-R	GCAGTGTGTCCGGCTGTACT
GAPDH-F	GGTTACCAGGGCTGCCTT
GAPDH-R	GAGTCATACTGGAACATGT

## Data Availability

The data used to support the findings of this study are included within the article.

## Additional Points

*Highlights*. (1) WTG ameliorated the articular inflammation, effectively inhibited the destruction of articular cartilage, and decreased the ratios of Th17 and Treg cells. (2) WTG effectively inhibited the levels of inflammatory cytokines and RNAKL and increased the levels of anti-inflammatory cytokines. (3) WTG might improve the imbalance of Th17/Treg cells in CIA rats by regulating the differentiation of Th17 and Treg cells.
